# Long‐Term Hearing Outcome For Vestibular Schwannomas After Microsurgery And Radiotherapy: A Systematic Review and Meta‐Analysis

**DOI:** 10.1002/ohn.910

**Published:** 2024-07-24

**Authors:** Antonio Daloiso, Diego Cazzador, Stefano Concheri, Giulia Tealdo, Elisabetta Zanoletti

**Affiliations:** ^1^ Department of Neuroscience DNS, Otolaryngology Section University of Padova Padova Italy

**Keywords:** acoustic neuroma, hearing loss, hearing preservation, meta‐analysis, microsurgery, radiosurgery, stereotactic radiotherapy, systematic review, vestibular schwannoma

## Abstract

**Objective:**

Hearing loss is a common symptom associated with vestibular schwannoma (VS), either because of the tumor's effects on the cochlear nerve or due to active treatments such as surgery or stereotactic radiosurgery (SRS). Treatment decisions for VS are based on factors including tumor size, hearing status, patient symptoms, and institutional preference. The study aimed to investigate long‐term auditory outcomes in VS patients undergoing active treatments with a hearing preservation intent.

**Data Sources:**

A systematic literature review was conducted following Preferred Reporting Items for Systematic Reviews and Meta‐Analyses guidelines, searching Scopus, Pubmed, and Web of Science databases from inception to January 2024.

**Review Methods:**

Studies meeting inclusion criteria, including a minimum 5‐year follow‐up and assessment of pre‐ and posttreatment hearing outcomes, were included. Pooled prevalence estimates for serviceable hearing after SRS and microsurgery were calculated using MetaXL software. Risk of bias assessment was performed with the Risk of Bias in Non‐randomized Studies of Interventions tool.

**Results:**

Nine studies met the inclusion criteria, with 356 patients included for analysis. The pooled prevalence of maintaining serviceable hearing after SRS at 10 years was 18.1% (95% confidence interval [CI]: 1.7%‐43.3%), with wide prediction intervals indicating variability in outcomes. Microsurgery demonstrated a higher prevalence of maintaining long‐term serviceable hearing, with a pooled estimate of 74.5% (95% CI: 63.5%‐84.1%).

**Conclusion:**

This systematic review underscores the importance of long‐term follow‐up in evaluating auditory outcomes in VS treatment. Despite the biases inherent to pretreatment patients selection, hearing preservation microsurgery for sporadic VS removal demonstrated favorable and stable long‐term serviceable hearing.

Vestibular schwannomas (VSs) are commonly defined as relatively rare tumors. However, recent studies demonstrated an annual incidence rate among all ages ranging between 3.0 and 5.2 VS per 100,000 person‐years.^1^, ^2^ The incidence of VS has been increasing in recent years due to advances in modern imaging techniques, particularly, magnetic resonance imaging. VSs are benign, slow‐growing tumors that originate from the vestibular portions of the eighth cranial nerves.^3^ Sporadic unilateral VS accounts for approximately 90% of cases,^4^ while bilateral VS is associated with neurofibromatosis Type 2. Although dizziness and tinnitus may occur, the most common presenting symptom is ipsilateral sensorineural hearing loss in more than 90% of cases.^4^ Hearing loss etiology can be classified as tumorigenic or iatrogenic. The former can not only be determined by the direct compression effect of the tumor on the cochlear nerve, but also caused by the activation of inflammatory pathways, immune mechanisms, and ototoxic factors secreted by the VS.^5^, ^6^, ^7^ The iatrogenic hearing impairment is the consequence of active tumor treatments (microsurgery or stereotactic radiosurgery [SRS]).

Multiple treatment options as observation, SRS, and microsurgery are available to manage a VS. Several tumor‐ and patients'‐related factors come into play in treatment selection like tumor size, hearing, facial nerve status, patient's symptoms, age, and comorbidities. Institutional preference might also play a role, but patients' preference and a shared decision‐making represent an advisable policy of management. In cases of small VS with good hearing function, an attempt to perform hearing preservation surgery (HPS) may be considered.^8^ This approach assumes that it provides the best chances of long‐term hearing preservation for patients.^9^ However, for short and midterm outcomes, conservative therapies such as SRS and observation may offer better results. The issue is debated, since reporting long‐term results is, particularly, relevant in nonsurgical treatment, where *success* does not mean removal of the tumor but control of growth, and both efficacy and side effects are to be considered in later years.

The purpose of this study was to investigate long‐term auditory outcomes in VS submitted to active treatments such as SRS or microsurgery with hearing preservation intent. A systematic literature review was conducted on this topic and a quantitative analysis of prevalence was performe for long‐term maintained serviceable hearing after treatment.

## Materials and Methods

### Protocol Registration

This systematic review protocol was registered in March 2023 before study commencement in the International Prospective Register of Systematic Reviews (PROSPERO, registry number CRD42023400669).

### Search Strategy

A systematic literature review was conducted according to the Preferred Reporting Items for Systematic Reviews and Meta‐Analyses (PRISMA) recommendations.^10^ The PRISMA Checklist is available as Supplemental Data S1, available online. The electronic databases Scopus, Pubmed, and Web of Science were searched from database inception to January 13, 2024. The search strategy combined various medical subject headings and text words for surgery, radiation therapy, hearing preservation, and audio‐vestibular symptoms (see Supplemental Data S2, available online). The reference lists of all the included articles were thoroughly screened to find other relevant articles. References were exported to the Zotero bibliography manager (v6.0.10, Center for History and New Media, George Mason University, Fairfax, VA, USA). After duplicate removal, 2 reviewers (A.D. and D.C.) independently screened all titles and abstracts and then evaluated the full texts of the eligible articles based on the inclusion criteria. Any disagreement was resolved through discussion with all authors to reach a consensus.

### Selection Criteria

Studies were deemed eligible when the following inclusion criteria were met: (i) peer‐reviewed studies on sporadic VS treated either with SRS or microsurgery with a hearing preservation intent; (ii) audiological follow‐up ≥5 years for each patient included. When raw data on follow‐up were not available, studies with a lower follow‐up range value of ≥5 years were selected; (iii) studies including at least 10 patients; (iv) available pretreatment and long‐term posttreatment hearing outcomes, possibly assessed through either Gardner‐Robertson (GR),^11^ or American Academy Otolaryngology–Head and Neck Surgery (AAO‐HNS) classifications.^12^ Pure tone average (PTA) ≤ 50 dB and speech discrimination score (SDS) ≥ 50% defined *serviceable hearing*, that is, GR or AAO‐HNS classes A or B. We defined long‐term auditory outcome as audiometric results available at least 5 years after treatment.

Criteria for exclusion were as follows: (i) studies dealing with neurofibromatosis type‐2 patients; (ii) recurrent VS or residual tumors, treated both with SRS and/or microsurgery; (iii) lack of relevant data in terms of hearing assessment and follow‐up; (iv) nonoriginal studies (ie, reviews, recommendations, letters, editorials, or book chapters); (v) non‐English studies. The papers were thoroughly screened for duplicates.

### Data Extraction and Quality Assessment

Extracted data were collected in an electronic database including first author, year of publication, country of origin, study design, enrollment period, type of treatment, total sample size, number of patients with pretreatment serviceable hearing, number of patients with serviceable hearing after treatment, preoperative audiological status, posttreatment long‐term audiological outcomes, follow‐up.

The quality of the eligible studies was categorized as Poor, Fair, and Good, in agreement with the National Institutes of Health quality assessment tool for Observational Cohorts and Cross‐Sectional Studies (https://www.nhlbi.nih.gov/health-topics/study-quality-assessment-tools, accessed on January 13, 2024).^13^ Two reviewers (A.D. and D.C.) independently evaluated the papers, and any disagreement was resolved by consensus. Risk of bias assessment for nonrandomized studies was performed with the Risk of Bias in Non‐randomized Studies of Interventions (ROBINS‐I) tool.^14^ Importantly, ROBINS‐I bias assessments are made based on the comparison between a given study and a theoretical randomized‐controlled trial with ideal design for the study question—the latter of which represents the standard for a “low‐risk” study.

### Statistical Analysis

To produce pooled estimates for the prevalence of serviceable hearing after VS treatment (SRS or microsurgery) the software MetaXL version 5.3 (EpiGear International Pty Ltd) was used. Initially, the observed prevalence for each study was transformed using the Freeman‐Tukey double arcsine transformation to stabilize the variance.^15^, ^16^ DerSimonian‐Laird model with random effects generated the overall pooled estimates with 95% confidence interval (95% CI).^17^ Meta‐analysis results were summarized graphically using forest plots, including the individual papers' proportion, pooled proportions with corresponding 95% CI, and study weights. Variance in true effects was assessed by *I* ^2^ statistics.^18^ 95% prediction intervals (95% PIs) of the pooled effect size were computed as measure of index of dispersion of the true effect^19^, ^20^ by using the free Prediction Intervals Program (www.meta-analysis.com accessed on January 13, 2024).^21^ Publication bias was graphically investigated with the Doi plot and quantified with the Luis Furuya‐Kanamori (LFK) index, which was interpreted as no asymmetry for values within ±1, minor asymmetry for values >±1, and within ±2, major asymmetry for values >±2.^22^

## Results

### Search Results and Study Selection

**Figure 1:**
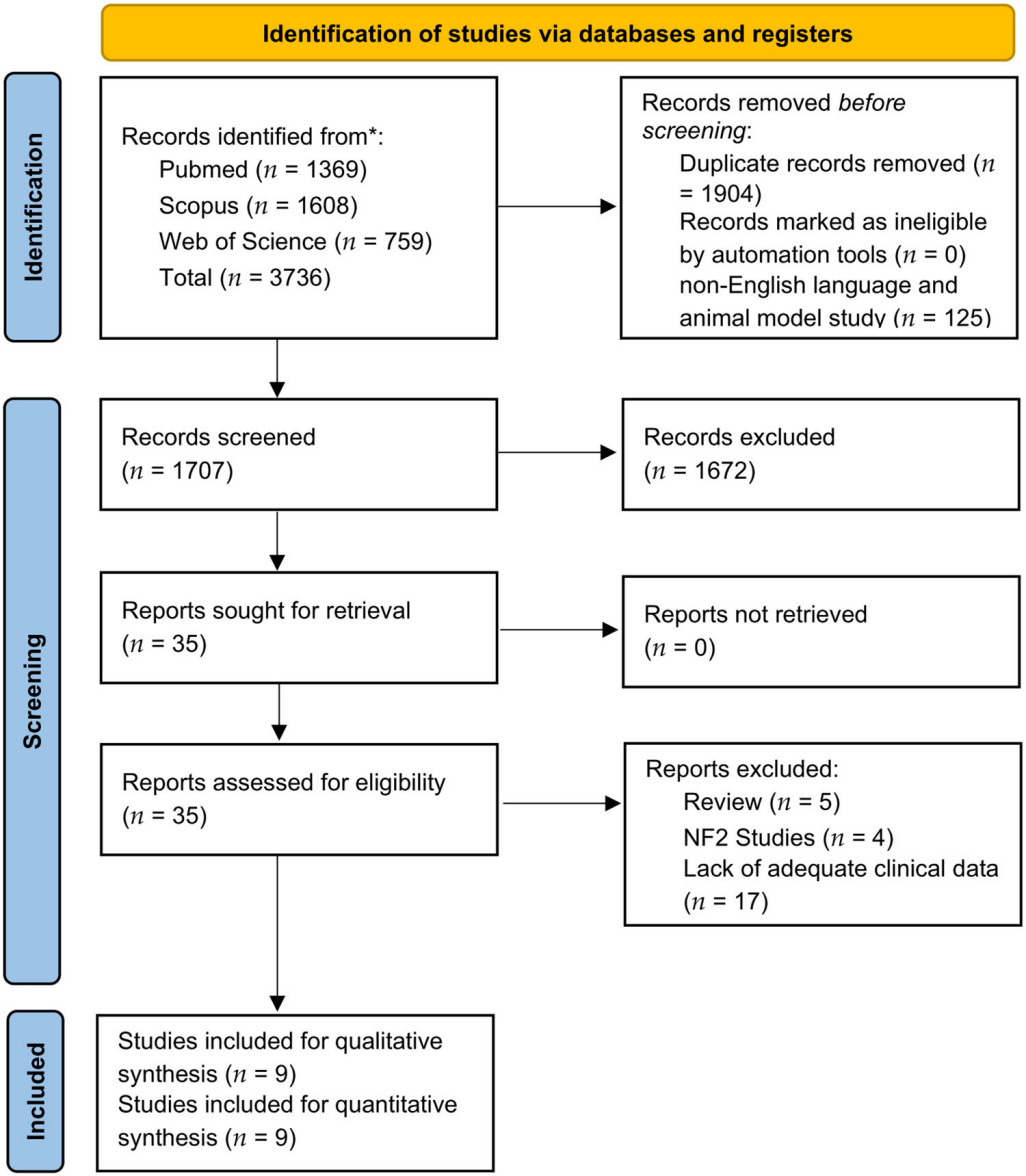
Flowchart depicting the record selection and article inclusion/exclusion process.

A total of 3736 titles were collected from our literature search. After duplicates and non‐English studies removal, 1672 records were discarded according to the inclusion/exclusion criteria, and 35 articles relevant to the topic were retrieved and assessed for eligibility. Finally, 9 studies were included for qualitative and quantitative analysis.^23^, ^24^, ^25^, ^26^, ^27^, ^28^, ^29^, ^30^, ^31^ A detailed flowchart of the search process is shown in Figure 1.

### Quality of Studies Assessment and Risk of Bias Assessment

In accordance with the National Institutes of Health quality assessment tool for Observational Cohorts and Cross‐Sectional Studies,^13^ 1 study was deemed of Good quality (11.1%), 7 Fair (77.8%), and only 1 (11.1%) were classified as Poor, due to the lack of reporting information on the series' features (see Supplemental Data S3, available online).

Risk of bias summaries for nonrandomized studies is given in Supplemental Data S4, available online. One study was assessed as “critical risk,” 6 were scored as “serious risk,” primarily due to confounding and participants selection. Finally, 2 studies were classified as “moderate risk.”

### Characteristics of the Included Studies

Eight studies included in the qualitative analysis had an observational retrospective design,^24^, ^25^, ^26^, ^27^, ^28^, ^29^, ^30^, ^31^ while one had a prospective design.^23^ Findings of the retrieved articles are discussed in dedicated sections, and data on patients' demographics, study design, tumor treatment, and hearing results are reported in Table 1.

**Table 1:** Characteristics of the Included Studies

References	Country	Enrollment period	Treatment	Total number of patients	Patients with pretreatment serviceable hearing	Pretreatment hearing status according to AAO‐HNS and/or GR classifications	Pretreatment PTA and SDS^a^
Friedman et al^26^	United States	1990‐1995	MS	119	NR	NR^b^	PTA 26.3 ± 15.1 SDS 91 ± 10
Chee et al^31^	Canada	1978‐1997	MS	30	29	Class A^b^ 17 (56.7%) Class B^b^ 12 (40%)	PTA 25 ± 14 SDS 88 ± 11.6
Woodson et al^27^	United States	1994‐2007	MS	49	46	Class A^b^ 31 (67.4%) Class B^b^ 15 (32.6%) NR^c^	PTA 27 ± 12 SDS 89 ± 13
Mazzoni et al^28^	Italy	1985‐2006	MS	200	194	NR^b^	NR
Roos et al^23^	Australia	1993‐2000	SRS	44	28	NR	NR
Carlson et al^24^	United States	1997‐2002	SRS	56	44	Class A^b^ 25 (57%) Class B^b^ 19 (43%)	PTA 27 ± 11.7 (2.5‐50) SDS 88.9 ± 13.2 (91.5‐100)
Quist et al^29^	United States	1998‐2009	MS	57	49	NR^b^	PTA 23 (1‐50) SDS 97 (76‐100)
Maksimoski et al^25^	United States	1998‐2019	SRS	304 total 133 with available audiometric data	90	Class A^b^ 43 (32%) Class B^b^ 47 (35%) GR I^c^ 44 (32%) GR II^c^ 46 (35%)	NR
Park et al^30^	Korea	1997‐2004	SRS	106	56	GR I^c^ 32 (30.2%) GR II^c^ 24 (22.6%)	NR

PTA is calculated in dB, SDS as percentage.

Abbreviations: AAO‐HNS, American Academy Otolaryngology‐Head and Neck Surgery; GR, Gardner‐Robertson; MS, microsurgery; NR, not reported; PTA, pure tone average; SDS, speech discrimination score; SRS, stereotactic radiosurgery.

^a^ Mean ± standard deviation (range). Calculated for patients with pretreatment serviceable hearing.

^b^ AAO‐HNS classification.

^c^ GR classification.

Studies were published between 2003 and 2021, encompassing patients treated from 1978 to 2019. Among the 965 patients of the included studies, pretreatment serviceable hearing was present in 536. Long‐term posttreatment audiometric data were available for 356 patients, of which 218 received SRS and 138 were submitted to microsurgical VS resection. The audiological follow‐up ranged between 60 and 264 months. The AAO‐HNS classification was adopted for assessing hearing in 4 studies,^24^, ^26^, ^28^, ^29^, ^31^ and the GR classification in 1.^30^ Two studies reported hearing status classified according to both systems,^25^, ^27^ while Roos et al did not adopt any specific hearing classifications.^23^

As for SRS studies,^23^, ^24^, ^25^, ^30^ the characteristics and audiological long‐term outcomes are reported in Table 2. Three of the included studies utilized the Gamma‐Knife,^24^, ^25^, ^30^ and one the linear accelerator technique,^23^ with a mean margin dose ranging from 8 to 16 Gy. Two studies reported on the mean cochlear dose.^24^, ^30^ As only Carlson et al,^24^ reported pretreatment mean PTA and SDS for patients with serviceable hearing and long‐term audiological assessment, the pooled data for the SRS study group were not calculated. All the included studies reported long‐term audiological outcomes at 10‐year follow‐up, and additionally, 2 studies defined outcomes at 5‐ and 7‐year follow‐up, as well.^23^, ^25^

**Table 2:** Characteristics and Audiological Long‐Term Outcomes of the SRS Studies Included

References	SRS technique	Mean marginal dose, Gy	Mean cochlear dose, Gy	Patients with available long‐term follow‐up	5 y posttreatment serviceable hearing rates	5 y actuarial serviceable hearing (95% CI)	7 y posttreatment serviceable hearing rates	7 y actuarial serviceable hearing (95% CI)	10 y posttreatmentserviceable hearing rates	10 y actuarial serviceable hearing (95% CI)
Roos et al^23^	LINAC	12‐14	NR	28	NR	57% (38‐74)	NR	NR	29% (8/28)	24% (11‐44)
Carlson et al^24^	GK	12 in 41 patients 13 in 3 patients	5.0 ± 1.8 (2.0‐8.3)	44	SH 47.7% (21/44)	48% (35‐65)	SH 36.4% (16/44)	38% (26‐56)	9.1% (4/44)	23% (13‐41)
Maksimoski et al^25^	GK	11.0‐16.0	NR	90	SH 23.3% (21/90) PTA 62.6 (53.5‐71.9) SDS 39.5 (28.6‐50.3)	NR	SH 13.3% (12/90) PTA 68.1 (55.2‐81.0) SDS 38.3 (23.6‐53‐0)	NR	SH 4.1% (4/90) PTA 76.9 (60.0‐93.8) SDS 29.3 (12.9‐45.6)	NR
Park et al^30^	GK	12.5 (8‐15)	3.60 ± 2.60	56	NR	NR	NR	NR	SH 46.4% (26/56 patients)	NR

PTA is calculated in dB, SDS as percentage.

Abbreviations: GK, Gamma Knife; LINAC, linear accelerator; NR, not reported; PTA, pure tone average; SDS, speech discrimination score; SH, serviceable hearing; SRS, stereotactic radiosurgery.

Regarding MS studies (Table 3),^26^, ^27^, ^28^, ^29^, ^31^ 3 studies treated patients via a middle cranial fossa (MCF) approach,^26^, ^27^, ^29^ and 2 via a retrosigmoid (RS) corridor.^28^, ^31^ When the data were available, 318 patients presented with preoperative serviceable hearing, and 188 (59.1%) retained serviceable hearing in the early postoperative period. According to the inclusion criteria, 138 patients with early postoperative serviceable hearing and long‐term follow‐up were considered for quantitative analysis. Pooled preoperative mean PTA and SDS were 24.9 dB (range: 1‐50) and 91.9% (range: 56‐100), respectively. These results were calculated based on the line data available in the studies.^27^, ^29^, ^31^

**Table 3:** Characteristics Audiological Long‐Term Outcomes of the Microsurgery Studies Included

References	Microsurgical approach	Patients with preserved postoperative hearing	Patients with early postoperative serviceable hearing and available long‐term follow‐up	Follow‐up for the included patients, mo	Early postoperative hearing assessment^a^	Last postoperative serviceable hearing rates	Last postoperative hearing assessment^a^
Friedman et al^26^	MCF	NR	23	≥60	PTA 48.7 ± 19.3 SDS 80.2 ± 28	16/23 (69.6%)	PTA 48.6 ± 19.6 SDS 70 ± 30.8
Chee et al^31^	RS	24/29 (82.7%)	19	113.8 ± 50.7^a^ (65‐264)	PTA 27.5 ± 15.5 SDS 89.0 ± 10.1	14/19 (73.7%)	PTA 45.8 ± 9.1 SDS 81.9 ± 22.1
Woodson et al^27^	MCF	43/46 (93.5%)	26	92.5 ± 26.3^a^ (62‐163)	PTA 34.1 ± 11.6 SDS 86.2 ± 11.4	16/26 (61.5%)	PTA 48.2 ± 18.0 SDS 83.7 ± 20.1
Mazzoni et al^28^	RS	94/194 (48.5%)	54	168^a^ (72‐252)	NR	47/54 (87%)	NR
Quist et al^29^	MCF	27/49 (55.1%)	16	≥60	PTA 33.0 ± 14.1 SDS 95.4 ± 9.8	12/16 (75%)	PTA 41.0 ± 18.8 SDS 91.7 ± 11.6

PTA is calculated in dB, SDS as percentage.

Abbreviations: HPS, hearing preservation surgery; MCF, middle cranial fossa; MS, microsurgery; NR, not reported; PTA, pure tone average; RS, retrosigmoid; SDS, speech discrimination score; SH, serviceable hearing.

^a^ Mean ± standard deviation (range).

Two studies reported minimum follow‐up as ≥60 months,^26^, ^29^ and the mean follow‐up for the other 3 studies was 124.8 months (range: 62‐264).^27^, ^28^, ^31^ The pooled mean postoperative PTA and SDS calculated in the early postoperative period were 35.8 dB (range: 27.5‐48.7) and 87.7% (range: 80.2‐95.4), respectively. At last follow‐up available, pooled mean PTA was 45.9 dB (range: 41‐48.6) and SDS 81.9% (range: 71‐93).

### Long‐Term Prevalence of Serviceable Hearing After SRS

**Figure 2:**
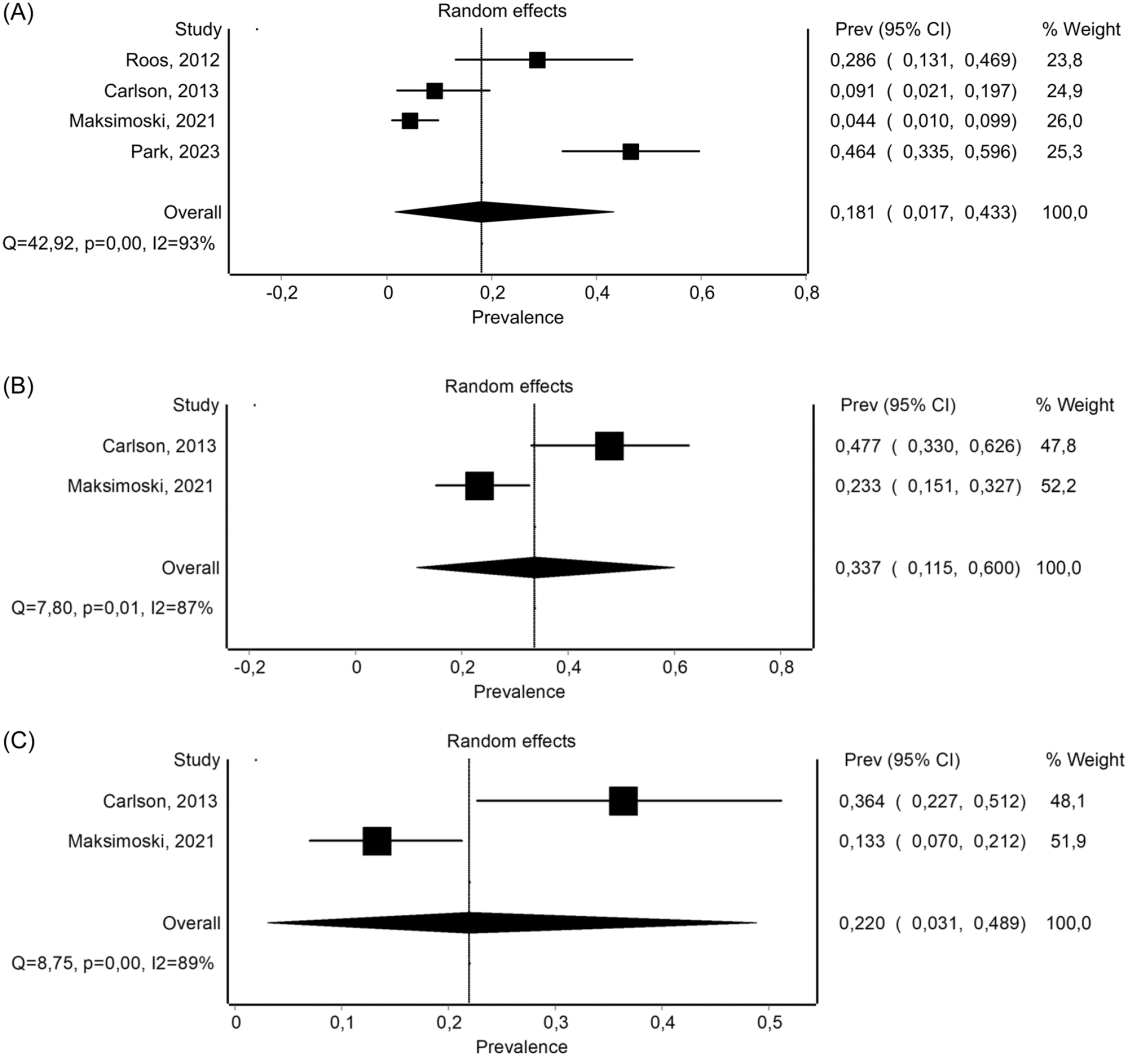
Forest Plot for stereotactic radiosurgery. Overall effects estimate on long‐term hearing (A), at 5‐year follow‐up (B) and 7‐year follow‐up (C). CI, confidence interval.

The pooled prevalence of serviceable hearing after SRS for VS is represented as a forest plot in Figure 2. The random‐effects pooled estimated prevalence of maintaining serviceable hearing 10 years after SRS was 18.1% (95% CI: 1.7%‐43.3%) with a wide range of 95% PI between 1.0% and 88.0% (Supplemental Data S5, available online). Calculated variance in true effects was *I* ^*2*^ = 93%. Doi plot for publication bias showed minor asymmetry, as confirmed by LFK index = 1.85 (Supplemental Data S6, available online). At 5‐ and 7‐year follow‐up after SRS, the pooled prevalences of serviceable hearing were 33.7% (95% CI: 11.5%‐60.0%), and 22.0% (95% CI: 3.1%‐48.9%), respectively. Corresponding 95% PIs were not calculated for these pooled proportions, as the number of included studies was less than 3 (forest plots are shown in Figure 2).

### Long‐Term Prevalence of Serviceable Hearing After Microsurgery

**Figure 3:**
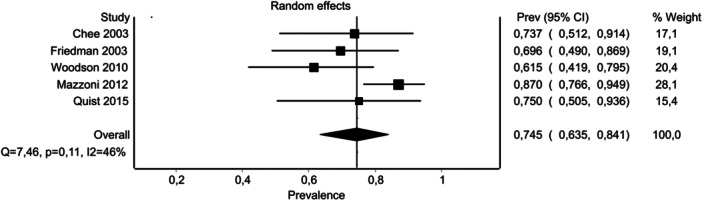
Forest Plot for microsurgery—effects estimates on long‐term hearing. CI, confidence interval.

The pooled prevalence of serviceable hearing after microsurgery for VS is represented as a forest plot in Figure 3. The random‐effects pooled estimated prevalence of maintaining serviceable hearing at last follow‐up was 74.5% (95% CI: 63.5%‐84.1%), with a range of 95% PI between 49.0% and 90.0% (Supplemental Data S5, available online). Calculated variance in true effects was *I* ^*2*^ = 46.4%. Doi plot for publication bias showed no asymmetry, as confirmed by LFK index = −0.70 (Supplemental Data S6, available online).

## Discussion

### Summary of Findings

In this study, we aimed to investigate the long‐term prevalence of serviceable hearing following SRS and microsurgery. The meta‐analysis revealed that the prevalence of maintaining serviceable hearing after SRS for VS was 18.1% at the 10‐year mark, indicating that most patients do not maintain posttreatment functional long‐term hearing. Notably, the wide 95% PI ranging from 1.0% to 88.0% underscores the considerable variability in hearing outcomes observed across studies. At the 5‐ and 7‐year follow‐up intervals, the pooled prevalences of serviceable hearing were 33.7% and 22.0%, respectively. While these proportions suggest a higher likelihood of hearing preservation compared to the 10‐year mark, the wide 95% CI reflected the limited number of the included studies.

In contrast to what observed after SRS, long‐term audiological outcomes after microsurgery demonstrated a substantially higher long‐term prevalence of maintaining serviceable hearing. The pooled estimate of 74.5% suggests that a significant majority of patients who underwent successful microsurgical VS removal with hearing preservation, retained long‐term functional hearing. The narrower 95% PI between 49.0% and 90.0% indicates less variability in outcomes compared to SRS. Furthermore, analyses for publication bias and sensitivity revealed no significant asymmetry or variation, indicating robustness and consistency in the findings across studies.

### Comparison to Other Studies

Considering the different treatment options for VS, observation, SRS, and microsurgery diverge in the long‐term durability of preserved hearing. Observation provides good short‐ and mid‐term results, but predictably poor long‐term hearing preservation rates.^32^ SRS is unlikely to cause immediate hearing loss, yet many patients experience progressive hearing loss over time.^33^ The reported SRS hearing preservation rates are heterogeneous between studies. Many systematic reviews have been published on this topic, showing at mid‐term follow‐up hearing preservation rates ranging between 41% and 88%.^34^, ^35^, ^36^ Analogously, Tsao et al^37^ reported 5‐year actuarial hearing preservation rates ranging between 41% and 79%. However, overall long‐term follow‐up data shows a decline in hearing preservation over time of 49%, 24%, and 12% at 5‐, 10‐ and 15‐year postradiation, respectively, in Watanabe et al^38^ and 59.4% at median follow‐up of 6.7 years (2‐23 years) in Ballosier et al.^33^

Microsurgery has the highest risk of hearing loss in the early posttreatment period. Sughrue et al^39^ sought to evaluate the rates of hearing preservation at any point after microsurgical resection, finding an overall postoperative hearing preservation rate of 52%. In their meta‐analysis, Ahsan et al^40^ attempted to evaluate long‐term hearing preservation following HPS and showed hearing durability rates between 60% and 92% at last follow‐up (mean follow‐up 104.3 months, range: 14‐264). Hunt et al^41^ evidenced that 58% of patients with preoperative serviceable hearing retained it at last audiological assessment (mean follow‐up for the included studies was 52.5 months). Furthermore, they stated that, conversely to SRS, prolonged follow‐up was not associated with worsened hearing preservation in microsurgery. Considering the importance of long‐term follow‐up to define the evolution of hearing in VS treatment, Golfinos et al^42^ outlined that SRS was superior to microsurgery in preserving hearing due to the shorter follow‐up time of the SRS patients' group.

To date, few studies have compared the outcomes of different VS management strategies. In 2000, a meta‐analysis by Kaylie et al^43^ comparing microsurgery and SRS reported no significant difference (*P* = .82) in hearing preservation, with rates of 44% in both groups. Different results were reported by Maniakas and Saliba^44^ who observed overall useful hearing preservation rates in SRS and microsurgery patients of 70.2% and 50.3%, respectively. Finally, a systematic review by Aman et al^45^ evidenced that in all the studies included, SRS was superior to microsurgery in preserving hearing function. The following considerations must be addressed, though. In Kaylie et al,^43^ VS of up to 4 cm in diameter were included in the analysis, whereas the other 2 comparative studies focused on smaller‐size tumors excluding all VS larger than 2 and 2.5 cm, respectively.^44^, ^45^ In fact, tumor size has a considerable impact on HPS rates.^9^, ^46^, ^47^

### Clinical Impact

Data comparison considering hearing preservation after microsurgery and SRS is challenging to address. A detailed description of possibly homogeneous ways of tumor measurement and reporting outcomes is essential, considering the current heterogeneous methodologies in reporting hearing status. Several methods of PTA assessment, and different hearing evaluation scores are provided by the diverse hearing classifications available worldwide (to cite some of the mostly widespread: GR,^11^ AAO‐HNS,^12^ Tokyo,^48^ Sanna,^49^ and Word Recognition Score classifications^50^).

The identification of the best hearing‐preservation treatment between SRS and surgical options is influenced by case‐specific, tumors', and patients' factors. Hearing status and the possibility of hearing preservation with microsurgery or SRS are crucial considerations in the decision‐making process for patients. Among the factors influencing HPS outcomes, such as preoperative candidates' selection in terms of preoperative hearing and tumor size,^9^ the surgical technique plays a paramount role.

Considering microsurgery, MCF, and RS approaches are invariably contemplated for patients with preoperative serviceable hearing and small tumors. They provide exposure of the entire internal auditory canal up to the fundus and offer the possibility of hearing preservation.^51^ Several studies suggest that smaller tumors are easier to dissect from the nerves, being tumor size a prognostic factor for hearing preservation.^9^, ^52^ RS and MCF surgical approaches demonstrated comparable results in terms of hearing. In a 2012 retrospective study by Mazzoni et al,^28^ involving sporadic VS of any size treated with the RS approach and retrolabyrinthine meatotomy, overall hearing preservation was achieved in 97 out of 200 cases (48.5%). Notably, 87% of postoperative classes A + B (AAO‐HNS classification) cases demonstrated high long‐term hearing stability, with a deterioration rate of 13%. Similarly, in Chee et al^31^ a retrospective analysis of 30 cases surgically treated by RS approach evidenced a hearing preservation rate of 65.4% in patients with audiological follow‐up of ≥5 years. A 5‐year follow‐up study by Quist et al^29^ investigated patients undergoing MCF approach, focusing on 49 patients with AAO‐HNS Class A or B hearing preoperatively. Initial results showed 55% maintaining Class A/B hearing postsurgery, of which 75% maintained it over 5 years. Woodson et al^27^ demonstrated a 57% long‐term hearing preservation rate in small tumor removal through MCF surgery with a minimum 5‐year follow‐up. Friedman et al^26^ reviewed MCF surgeries from 1990 to 1995, reporting 61% of cases with postoperative preserved hearing, of which 70% maintained it for more than 5 years.

SRS has been identified as a potential alternative to microsurgery for patients with VS. Reported tumor control rates exceed 90% in some series.^33^, ^53^, ^54^ However, pretreatment tumor growth is a crucial factor to be defined before SRS commencement, especially in small VS that typically demonstrates no—or very slow growth. This might affect treatment outcomes in the literature, as demonstrated by Marston et al^55^ that evidenced a different tumor control rate according to pretreatment fast and slow growth (69% vs 97%, respectively). If the long‐term period evaluation is crucial to assess hearing stability after microsurgery, this becomes more evident considering nonsurgical treatments like SRS, where the effects of irradiation on hearing over time add up to that given by the natural history of the tumor. SRS demonstrated excellent short‐ and mid‐term results on hearing preservation. Nevertheless, at 10‐year follow‐up, reported rates ranged from 4.1% to 46.4%.^25^, ^30^ Pretreatment hearing status,^24^, ^25^, ^56^, ^57^ and tumor size^24^, ^56^, ^57^ have been identified as prognosticators for hearing preservation after SRS. Brainstem contact,^24^ and age under 60^57^ emerged as factors increasing the risk of developing nonserviceable hearing after SRS. Postattinic hearing decline mechanisms may include inner ear ischemia, posttreatment tumor expansion, and cochlear nerve demyelination.^56^

Of note, in the mid‐term, favorable audiological outcomes tend to favor SRS, while in the long‐term, the surgical option appears more advantageous. However, these conclusions are burdened by 2 fundamental biases: pretreatment patients' selection (considering hearing and tumor size) and surgical experience. Long‐term follow‐up beyond 10 years is crucial to better understand the durability of treatment effects, and the onset of late complications. Comparative studies evaluating the outcomes of active VS treatments as microsurgery and SRS in a well‐defined patient population would help in supporting clinical decision‐making.

### Strengths and Limitations

Several constraints are evident in the present investigation. First, the included studies consisted of retrospective case series, lacking in randomized clinical trials. Data reporting in the included studies was also extremely heterogeneous and often incomplete, specifically considering pretreatment tumor size or the accurate quantification of PTA and SDS. This aspect prevented us from conducting further subgroup analyses or pooled analysis of data. Additionally, the division of studies into subgroups based on treatment, although essential for comparing similar interventions, resulted in restricted sample sizes within each subgroup, some of which had a limited number of studies and cases. Despite these limitations in size, a comprehensive statistical analysis was conducted, recognizing that our findings could be bolstered by subsequent studies with larger sample sizes.

It is crucial to interpret the results with caution, given that many of the included studies exhibit a high risk of bias, as demonstrated by our formal risk of bias assessment using the ROBINS‐I tool.^14^ This assessment revealed significant heterogeneity among the studies, with variations in study design, patient populations, and outcome measures. Such heterogeneity, coupled with potential selection bias, substantially limits the robustness and generalizability of our findings. Specifically, the lack of detailed preoperative audiologic data and tumor size information further complicates the ability to draw definitive conclusions. While our study provides valuable insights into the long‐term hearing outcomes of microsurgery, these results should be interpreted within the context of the abovementioned limitations. Consequently, although short‐ and medium‐term data may support SRS, long‐term audiological results highlight a different trend.

To enhance our understanding of how each treatment modality compares to others, designing a multi‐institutional, prospective, randomized, controlled trial of primary SRS versus primary microsurgery approaches (ie, via MCF or RS approaches) is advocated.

## Conclusions

In conclusion, this systematic review and meta‐analysis highlighted the importance of long‐term follow‐up in evaluating the audiological outcomes of patients with sporadic VS treated either with microsurgery or SRS. The availability of data on long‐term hearing preservation rates following VS treatment is crucial in providing effective counseling for patients facing the possibilities of HPS, SRS, or observation. Despite the above‐mentioned biases inherent to the included studies, hearing preservation microsurgery for sporadic VS removal demonstrated favorable and stable long‐term serviceable hearing.

## Author Contributions

**Antonio Daloiso**, conceptualization, methodology, data curation, writing—original draft preparation, writing—review and editing; **Diego Cazzador**, conceptualization, methodology, data curation, writing—original draft preparation, writing—review and editing; **Stefano Concheri**, writing—review and editing, visualization; **Giulia Tealdo**, writing—review and editing, visualization; **Elisabetta Zanoletti**, conceptualization, writing—review and editing, supervision; All authors have read and agreed to the published version of the manuscript.

## Disclosures

### Competing interests

The authors declare that they have no conflict of interest.

### Funding source

Not applicable.

## Supporting information

Supporting information.

Supporting information.

Supporting information.

Supporting information.

Supporting information.

Supporting information.
