# Supplementary material for: Long‐Term Hearing Outcome For Vestibular Schwannomas After Microsurgery And Radiotherapy: A Systematic Review and Meta‐Analysis
Source: Otolaryngol Head Neck Surg. 2024 Jul 24;171(6):1670–81. doi: 10.1002/ohn.910 (PMC11605020; doi:10.1002/ohn.910)
Supplement: Supplementary file 2 — Supporting information. [file OHN-171-1670-s002.docx]

**Supplementary materials S2.** Seach strategy for each database.

**PUBMED**

("Vestibular schwannoma" OR "Acoustic neuroma") AND (Hearing OR "Hearing preservation" OR "Hearing outcome" OR Audiometric) AND (Microsurgery OR Retrosigmoid OR "Middle cranial fossa" OR Cyberknife OR "Gamma Knife" OR Radiotherapy OR "Stereotactic radiosurgery")

**SCOPUS**

( ( TITLE-ABS-KEY ( "Vestibular schwannoma" ) OR TITLE-ABS-KEY ( "Acoustic neuroma" ) ) ) AND ( ( TITLE-ABS-KEY ( hearing ) OR TITLE-ABS-KEY ( "Hearing preservation" ) OR TITLE-ABS-KEY ( "Hearing outcome" ) OR TITLE-ABS-KEY ( audiometric ) ) ) AND ( ( TITLE-ABS-KEY ( microsurgery ) OR TITLE-ABS-KEY ( retrosigmoid ) OR TITLE-ABS-KEY ( "Middle cranial fossa" ) OR TITLE-ABS-KEY ( cyberknife ) OR TITLE-ABS-KEY ( "Gamma Knife" ) OR TITLE-ABS-KEY ( radiotherapy ) OR TITLE-ABS-KEY ( "Stereotactic radiosurgery" ) ) ) AND ( LIMIT-TO ( SUBJAREA , "MEDI" ) ) AND ( LIMIT-TO ( LANGUAGE , "English" ) OR LIMIT-TO ( LANGUAGE , "German" ) ) AND ( LIMIT-TO ( SRCTYPE , "j" ) )

**WEB OF SCIENCE**

TI=("Vestibular schwannoma" OR "Acoustic neuroma") OR AB=("Vestibular schwannoma" OR "Acoustic neuroma") AND TI=(Hearing OR "Hearing preservation" OR "Hearing outcome" OR Audiometric) OR AB=(Hearing OR "Hearing preservation" OR "Hearing outcome" OR Audiometric) AND TI=(Microsurgery OR Retrosigmoid OR "Middle cranial fossa" OR Cyberknife OR "Gamma Knife" OR Radiotherapy OR "Stereotactic radiosurgery") OR AB=(Microsurgery OR Retrosigmoid OR "Middle cranial fossa" OR Cyberknife OR "Gamma Knife" OR Radiotherapy OR "Stereotactic radiosurgery")
