# Supplementary material for: Long‐Term Hearing Outcome For Vestibular Schwannomas After Microsurgery And Radiotherapy: A Systematic Review and Meta‐Analysis
Source: Otolaryngol Head Neck Surg. 2024 Jul 24;171(6):1670–81. doi: 10.1002/ohn.910 (PMC11605020; doi:10.1002/ohn.910)
Supplement: Supplementary file 3 — Supporting information. [file OHN-171-1670-s004.docx]

**Supplementary materials S3.** Quality assessment of the included studies.

| **Author, Year** | **Quality** |
| --- | --- |
| Friedman et al., 2003 [26] | Fair |
| Chee et al., 2003 [31] | Fair |
| Woodson et al., 2010 [27] | Fair |
| Mazzoni et al., 2012 [28] | Fair |
| Roos et al., 2012 [23] | Poor |
| Carlson et al., 2013 [24] | Fair |
| Quist et al., 2015 [29] | Fair |
| Maksimoski et al., 2021 [25] | Good |
| Park et al., 2023 [30] | Fair |
