# Supplementary figures and images for: Long‐Term Hearing Outcome For Vestibular Schwannomas After Microsurgery And Radiotherapy: A Systematic Review and Meta‐Analysis
Source: Otolaryngol Head Neck Surg. 2024 Jul 24;171(6):1670–81. doi: 10.1002/ohn.910 (PMC11605020; doi:10.1002/ohn.910)

**Risk of bias assessment for non-randomised studies (ROBIN-I tool)**

**
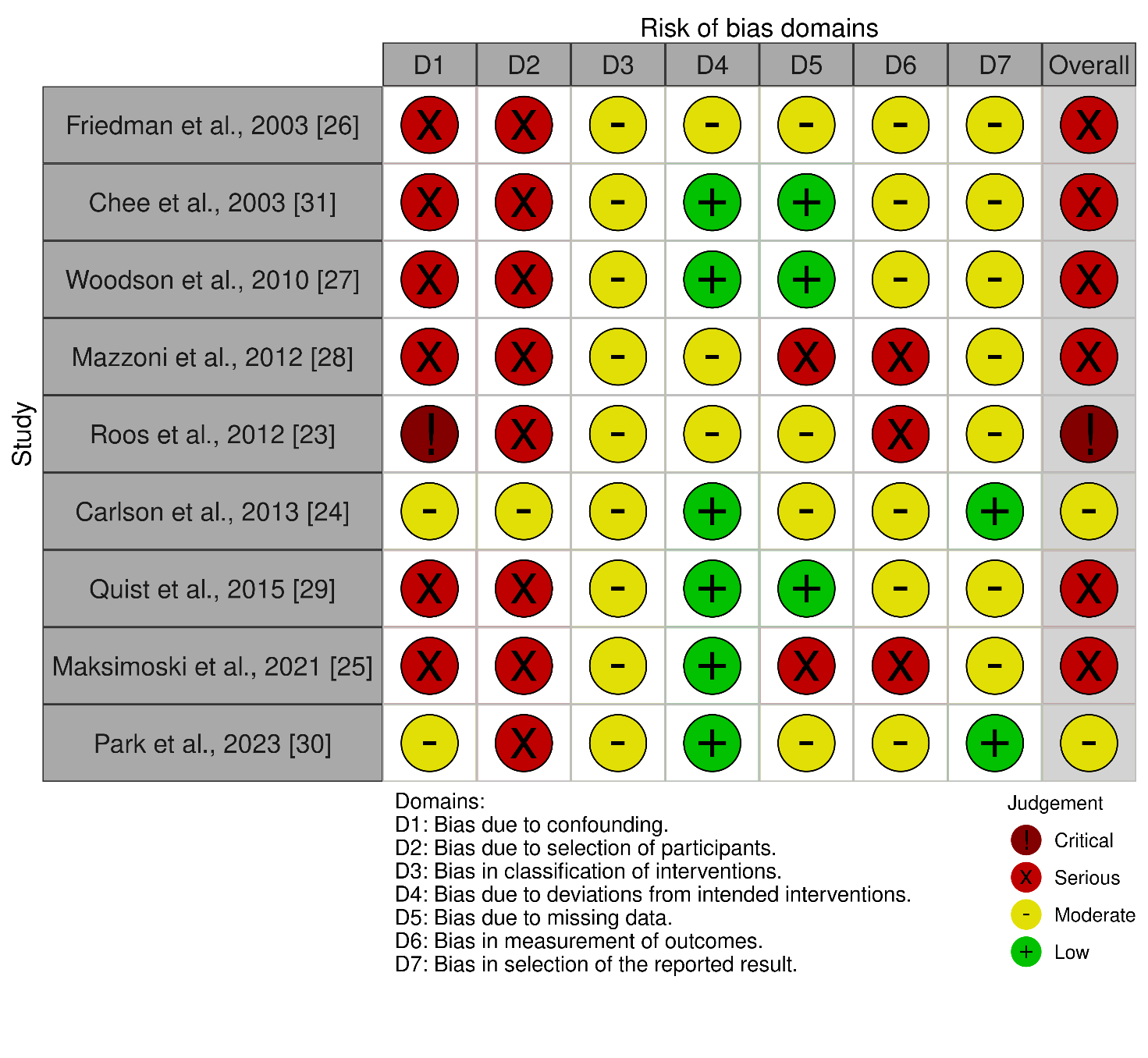
**

Supplement: Supplementary file 4 — Supporting information. [file OHN-171-1670-s003.docx]

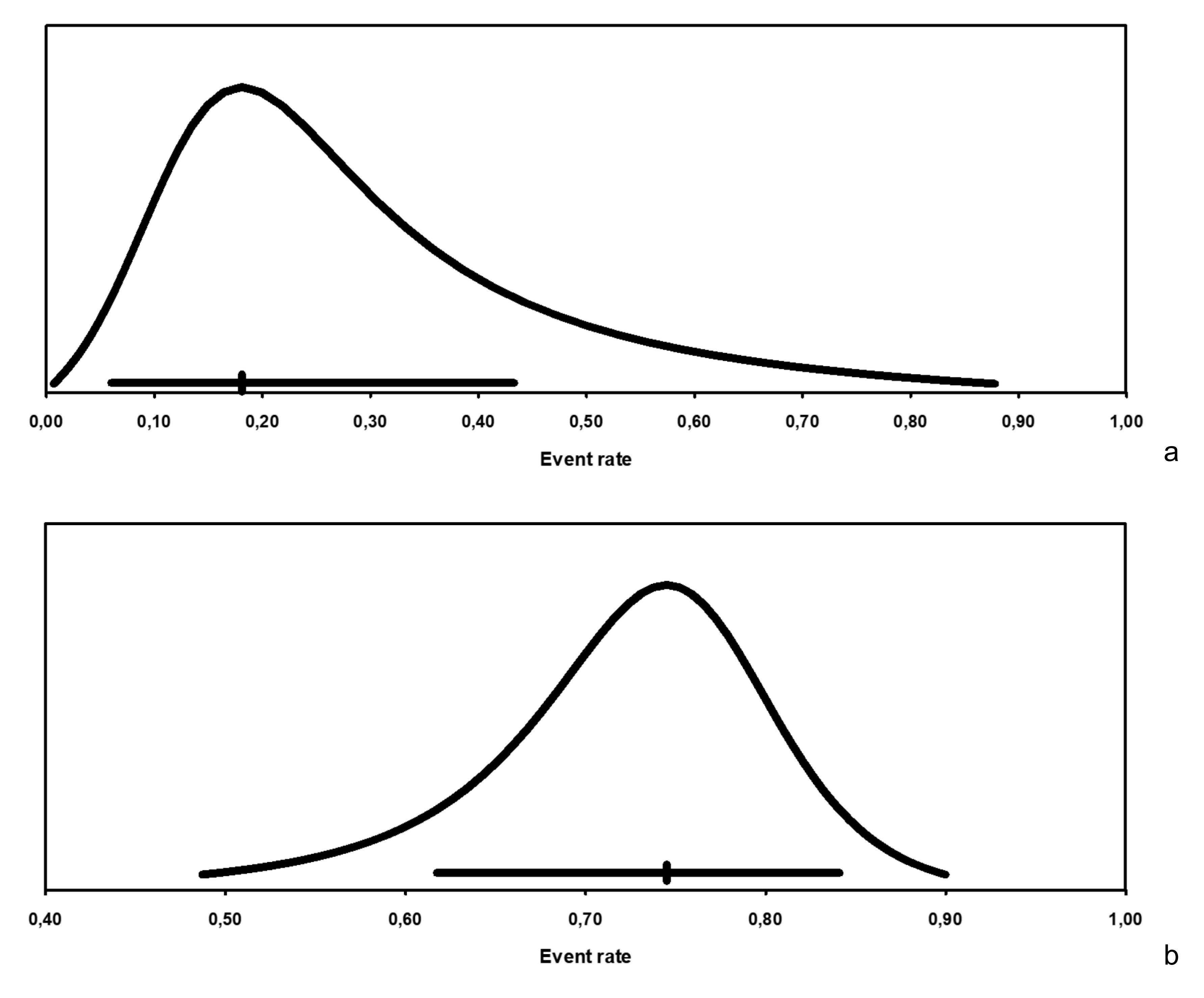

Supplement: Supplementary file 5 — Supporting information. [file OHN-171-1670-s001.tif]

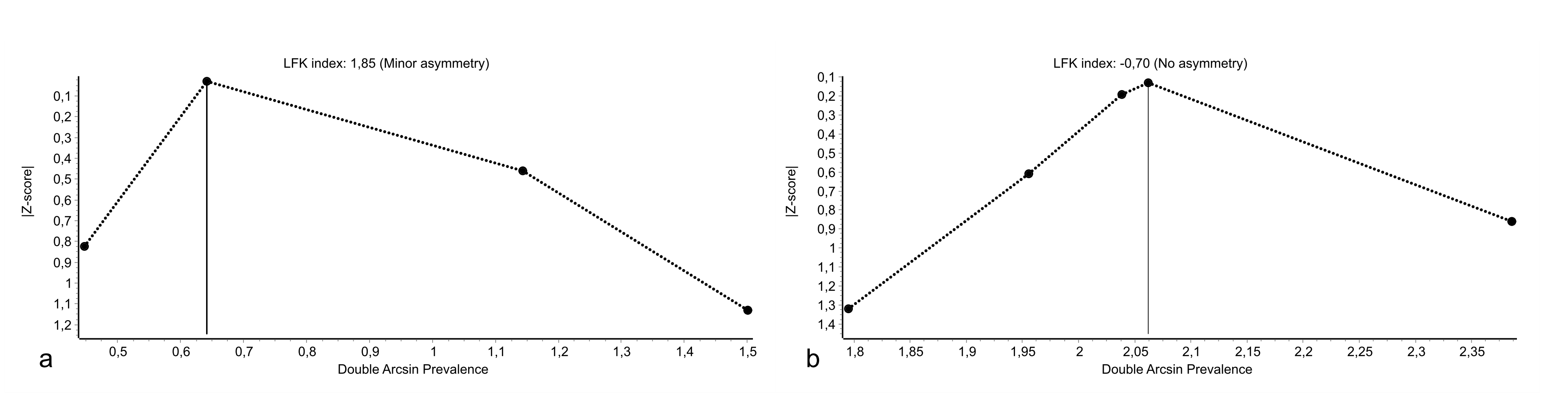

Supplement: Supplementary file 6 — Supporting information. [file OHN-171-1670-s006.tif]
